# Supplementary material for: Regulation of Intracellular Reactive Oxygen Species Levels after the Development of Phallus rubrovolvatus Rot Disease Due to Trichoderma koningii Mycoparasitism
Source: J Fungi (Basel). 2023 Apr 28;9(5):525. doi: 10.3390/jof9050525 (PMC10219376; doi:10.3390/jof9050525)
Supplement: Supplementary file 1 [file jof-09-00525-s001.zip › jof-2328101-supplementary.pdf]

Table S1. GenBank accession numbers of taxa used in phylogenetic analyses.

Species name	Strain number	GenBank accession number	
		**ITS**	**EF1-α**
Protocrea farinosa	CBS 121551	MH863119	EU703889
Protocrea pallida	CBS 299.78	MH861137	EU703900
T. achlamydosporum	YMF 1.06226*	MN977791	MT070156
T. afarasin	CBS 130755*/GJS 99-227	AY027784	AF348093
T. afroharzianum	CBS 124620*/GJS 04-186	FJ442265	FJ463301
T. amoenum	YMF 1.06210/YMF_1.6188	MN977802	MT070145
T. amoenum	YMF 1.06209	MN977801	MT070146
T. anaharzianum	YMF 1.00383*	MH113931	MH183182
T. anaharzianum	YMF 1.00241	MH262584	MH236493
T. asiaticum	YMF 1.00352*	MH113930	MH183183
T. asperellum	CBS 433.97=TR3*	AY380912	AY376058
T. atrobrunneum	T42	KX632515	KX632629
T. atroviride	TRS26	KJ786751	KJ786832
T. camerunense	CBS 137272*/GJS 99-230	AY027780	AF348107
T. endophyticum	CBS 130729*/DIS 217A	FJ442243	FJ463319
T. gamsii	G.J.S. 04-09	DQ315459	DQ307541
T. guizhouense	S278		KF134799
T. harzianum	CBS 226.95*	AJ222720	AF348101
T. harzianum	TRS55	KP009211	KP008803
T. hunanense	HMAS 248841*	KY687924	KY688039
T. inconspicuum	YMF 1.04623*	MK795993	MK795985
T. inhamatum	CBS 273.78*	FJ442680	AF348099
T. koningii	Hypo 51=CBS 119500	FJ860762	KC285594
T. koningiopsis	18ASMA001	MT520621	MT671922
T. lentiforme	CBS 100542*/GJS 98-6	AF469189	AF469195
T. lixii	CBS 110080*/G.J.S. 97-96	AF443920	AF443938
T. longiphialidicum	TC668		MF095879
T. longipilis	CBS 120953		FJ860643
T. longisporum	HMAS 248843*	KY687926	KY688043
T. ovalisporum	DIS 70a=CBS 113299*	AY380897	AY376037
T. petersenii	CBS 119507=Hypo 45	FJ860806	FJ860670
T. pollinicola	LC11682 = LF1542*	MF939592	MF939619
T. polypori	HMAS 248855*	KY687938	KY688058
T. propepolypori	YMF 1.06199/YMF_1.6176	MN977790	MT070157
T. propepolypori	YMF 1.06224*/YMF_1.6175	MN977789	MT070158
T. pseudoasiaticum	YMF 1.06200*/YMF_1.6178	MN977792	MT070155
T. pseudokoningii	G.J.S. 81-300	DQ083025	AY937429
T. pyramidale	CBS 135574*		KJ665699
T. reesei	G.J.S. 97-38	AJ004962	JN175603
T. rifaii	CBS 130745/DIS 337F	FJ442621	FJ463321
T. simile	YMF 1.06201*	MN977793	MT070154

T. simmonsii	CBS 130431*/G.J.S. 91-138	AF443917	AF443935
T. spirale	TRS111	KP009301	KP008963
T. subazureum	YMF 1.06207*	MN977799	MT070148
T. subuliforme	YMF 1.06204*	MN977796	MT070151
T. supraverticillatum	YMF 1.06208*/YMF_1.6186	MN977800	MT070147
T. tibetica	YMF 1.05583*	MK779177	MK779179
T. tomentosum	S23		KJ665759
T. velutinum	C.P.K. 298		KJ665769
T. virens	Gli39=CBS 249.59	AF099005	AF534631
T. viride	CBS 119325	DQ677655	DQ672615
T. atroviride	CBS 119499 = Hypo 326	FJ860726	FJ860611
T. koningiopsis	**NY120302**	**OP604600**	**OP620745**
T. asperellum	**NY120304H**	**OP604601**	**OP620746**
T. pollinicola	**PL21811-12-2**	**OP604602**	**OP620747**
T. pollinicola	**PL218116D**	**OP604603**	**OP620748**
T. lixii	**PL218119D**	**OP604604**	**OP620749**
T. pollinicola	**QX211008-2**	**OP604605**	**OP620750**
T. tomentosum	**XY101301**	**OP604606**	**OP620751**
T. tomentosum	**XY101314**	**OP604607**	**OP620752**
T. koningii	**PL110114**	**OP604608**	**OP620753**

The type and ex-type strains are indicated with * after the strain number.

Our 9 strains introduced in this study are indicated in bold.
